# Clinical, brain imaging and therapeutic evaluation of toxoplasma encephalitis in HIV-infected patients in Yaounde

**DOI:** 10.1186/1742-4690-9-S1-P145

**Published:** 2012-05-25

**Authors:** Annick Mélanie Magnerou, V Sini, P Ongolo-zogo, JY Fonsah, AK Njamnshi, L Kaptue

**Affiliations:** 1 Université des Montagnes, Douala, Cameroon

## Introduction

Toxoplasma encephalitis is very common in patients with AIDS. The diagnosis is based mainly on the response to medical treatment.

## Objectives

To evaluate clinical diagnosis, CT-scan findings, therapy and evolution of AIDS patients with toxoplasmic encephalitis.

## Material and methods

It was a descriptive cohort study with a prospective and a retrospective phase. Neurologic deficit was scored by using the NIHSS (National Institute of Health Stroke Scale).

## Results

Sixty consenting patients were recruited, the mean age was 38.7 ± 9.7 years. The F/M sex ratio was 1.6. The mean CD4 cell was 53.5 ± 42.6 /µl. The main presenting complaints were motor deficits (65 %), seizures (40 %), headaches (31.7 %), language and the speech disturbances (35 %). Signs of meningeal irritation and raised intracranial pressure were found in 21 % and 10 % of the cases respectively.

The brain lesions were all heterogeneous with contrast enhancement in 80.8 % of cases. Most lesions were supratentorial in 92.3 % of cases, and multilobar in 69.2 %of cases. Abscesses were multiple in 51.7 % of cases, and associated with brain herniation in 61.5 % and hydrocephalus in 30.8 %.

With adequate treatment, 61.7% had complete resolution and 13.3 % of the patients, had persistence of neurologic signs at the end of the intensive treatment period. The neurologic deficits improved by 50 % by the 7th day of treatment and even more by the 14th day. The most encountered treatment options with comparable outcomes were sulfadiazine-pyrimethamine in 61.7 % of the cases, followed by Trimethoprime-sulfamethoxazole in 31.1 %.

## Conclusion

Focal neurologic deficits of progressive onset, with or without headache and seizures, are the signs and symptoms that alert the suspicion of toxoplasma encephalitis in HIV-infected patients. Adequate treatment leads to improvement in neurologic deficits from the first week of treatment, measured with the NIHSS.

